# Ethnic differences in the adaptation rate of HIV gp120 from a vaccine trial

**DOI:** 10.1186/1742-4690-6-67

**Published:** 2009-07-15

**Authors:** Marcos Pérez-Losada, David Posada, Miguel Arenas, David V Jobes, Faruk Sinangil, Phillip W Berman, Keith A Crandall

**Affiliations:** 1 CIBIO, Centro de Investigação em Biodiversidade e Recursos Genéticos, Universidade do Porto, Campus Agrário de Vairão, 4485-661 Vairão, Portugal; 2 Departamento de Bioquímica, Genética e Inmunología. Universidad de Vigo, 36310 Vigo, Spain; 3 Presidio Pharmaceuticals, Inc., San Francisco, CA 94158, USA; 4 Global Solutions for Infectious Diseases, South San Francisco, CA 94080, USA; 5 Department of Biomolecular Engineering, University of California, Santa Cruz, CA 95064, USA; 6 Department of Biology, Brigham Young University, Provo, UT 84602, USA

## Abstract

Differences in HIV-1 gp120 sequence variation were examined in North American volunteers who became infected during a phase III vaccine trial using the rgp120 vaccine. Molecular adaptation of the virus in vaccine and placebo recipients from different ethnic subgroups was compared by estimating the *d*_N_/*d*_S _ratios in viruses sampled from each individual using three different methods. ANOVA analyses detected significant differences in *d*_N_/*d*_S _ratios among races (*P *< 0.02). gp120 sequences from the black individuals showed higher mean *d*_N_/*d*_S _ratios for all estimators (1.24–1.45) than in other races (0.66–1.35), and several pairwise comparisons involving blacks remained significant (*P *< 0.05) after correction for multiple tests. In addition, black-placebo individuals showed significantly (*P *< 0.02) higher mean *d*_N_/*d*_S _ratios (1.3–1.66) than placebo individuals from the other races (0.65–1.56). These results suggest intrinsic differences among races in immune response and highlight the need for including multiple ethnicities in the design of future HIV-1 vaccine studies and trials.

## Findings

More than 33 million people are currently infected with HIV-1, resulting in 2–3 million deaths every year. Natural immunity to the virus is virtually nonexistent; hence, the creation of a vaccine to combat this global pandemic is an international public-health priority [1,2]. In 2003, the results were released for a phase III HIV-1 vaccine efficacy trial conducted in North America and The Netherlands (VAX004) [3]. This study tested the efficacy of bivalent vaccines containing recombinant HIV-1 envelope glycoprotein 120 (rgp120) antigens, the major antigen on the surface of the virus [4]. Overall, the vaccine candidate did not seem to reduce the incidence of HIV-1 infection, but an interesting trend was noted in the analysis of the different self-described ethnic groups [white (non-Hispanic), blacks, Hispanic, Asian, and "others"]. When only the non-white volunteers (17% of the total study population) were considered, the vaccine seemed to confer a slight benefit (*P *= 0.012). After adjustment for multiple tests, this difference was not significant (*P *= 0.13) [3]. Despite a lack of statistical support, this is not a trivial result. If this trend was to be confirmed, it would imply that non-whites developed protective immunity to HIV-1 or, even more important, that rgp120 immunogens could protect against HIV infection under certain circumstances. Here we explored this possibility further.

HIV-1 evolution is driven, to a significant extent, by the immune response. If viruses isolated from non-white patients are in fact under a stronger selection pressure either because of genetic differences in the magnitude, specificity, or potency of the natural immune response, or because of differences in factors affecting virus replication, we should expect higher ratios of nonsynonymous (amino acid changing) to synonymous nucleotide substitutions (*d*_N_/*d*_S_) [5-8] than in viral samples isolated from vaccinated and placebo (non-vaccinated) white individuals.

To test whether levels of selection were significantly different between vaccinated and placebo individuals in different races, we analyzed 3 clones per individual from 345 infected North Americans from the VAX004 study (Table 1; data available at ). Full-length HIV-1 subtype B gp120 sequences were amplified as described in Gilbert et al. [9]. Since, as expected, viruses isolated from individuals from the same race did not form monophyletic groups [10], viral samples for each patient were analyzed separately. In each case, individual clones were aligned in MAFFT v5.7 [11], and *d*_N_/*d*_S _ratios were estimated using Nei and Gojobori's method [6] in SNAP [12], model M0 (one-ratio) in PAML v3.14 [13], and Fixed Effects Likelihood (FEL) with tree branch correction in HYPHY [14]. In the latter case, we took recombination into account by first detecting recombination breakpoints with GARD [15], and then estimating the *d*_N_/*d*_S _ratios independently for each fragment.

**Table 1:** Mean *d*_N_/*d*_S _estimates across patients in PAML, SNAP and HYPHY.

	White (291)	Hispanic (22)	Black (12)	Asian (5)	Other (15)
SNAP	0.709	0.750	1.240	0.818	0.659
PAML	0.839	0.826	1.411	1.346	0.751
HYPHY	0.949	0.729	1.453	0.720	1.202

Individuals analyzed are indicated between parentheses.

Mean *d*_N_/*d*_S _ratios across races and treatments were compared using ANOVA, linear models (*lm*) and pairwise t-tests. Because treating all non-whites as a single unit is unrealistic considering their own genetic differences [3], we tested for differences in selection pressure on a race-by-race basis. Multiple significance in the pairwise t-tests was corrected using the Benjamini and Hochberg's procedure [16].

The estimates of *d*_N_/*d*_S _obtained with SNAP, PAML and HYPHY were all significantly correlated among the different estimators used (correlation coefficient > 0.85; *P *< 0.001). Importantly, the mean *d*_N_/*d*_S _ratios varied across races (Table 1), and these differences were globally significant (ANOVA;*P *< 0.02) for SNAP and PAML estimates. Blacks (vaccinees and placebo combined) showed higher *d*_N_/*d*_S _ratios for all the estimators than individuals from other ethnicities (Table 1). Significant differences (pairwise t-tests; *P *< 0.05) between black and white, Hispanic and "others" viral samples were observed for all the estimators before corrections, but only the comparisons involving SNAP and PAML estimates remained significant after the Benjamini and Hochberg's adjustment (Table 2). The higher *d*_N_/*d*_S _ratios observed for blacks suggest that the rate of virus evolution is greater in this group than in other volunteers. Differences in immune response to HIV-1 infection have been pointed out by the rgp120 HIV Vaccine Study Group [3] as one of the potential factors to explain vaccine efficiency differences between white and non-white volunteers in the VAX004 trial. Ethnic differences in immune response have been also reported for other viruses such as the hepatitis C virus [17].

**Table 2:** Statistically significant comparisons of *d*_N_/*d*_S _estimates among races and race-treatments.

	Race		Race by treatment	
		
Method	ANOVA	Corrected pairwise t-tests	ANOVA	*lm *coefficient
SNAP	(0.011)	black vs hispanic (0.020) black vs other (0.013) black vs white (0.004)	(0.025)	black placebo (0.001)
PAML	(0.019)	black vs hispanic (0.047) black vs other (0.047) black vs white (0.033)		black placebo (0.016)
HYPHY				black placebo (0.015)

Values in parentheses are *P*-values.

Does the greater virus adaptive variation presumed in black participants reflect genetic differences in the intrinsic (no-preconditioned) immune response to HIV-1, or is it a consequence of the conditioned immune response induced by vaccination with rgp120? Comparison of vaccine and placebo recipients showed different results based on the *d*_N_/*d*_S _estimators used. No significant differences were observed among vaccinees, but significant differences (ANOVA; *P *= 0.025) in SNAP *d*_N_/*d*_S _ratios were detected among placebo individuals. Moreover, black-placebo patients showed significantly (*lm *coefficients; *P *< 0.02) higher mean *d*_N_/*d*_S _ratios (1.3, 1.38 and 1.66, for SNAP, PAML and HYPHY, respectively) than the other races (0.65–1.01, 0.84–1.14 and 0.73–1.56, respectively). These results might indicate that natural differences in the immune response may have increased viral rgp120 adaptation in blacks.

In North America, blacks correspond to 42% of all newly diagnosed HIV/AIDS cases, while white (non-Hispanic) and Hispanic individuals represent approximately 40% and 17%, respectively [18]. If more data including both placebo and vaccinated recipients confirm that selection pressure differs between viruses infecting these three races, deciphering the genetic determinants of these differences should become a public-health priority. Indeed, our results highlight the need for selecting a broader representation of volunteers, based on ethnicity, in the design of future HIV-1 vaccine studies and trials [19].

## Competing interests

The authors declare that they have no competing interests.

## Authors' contributions

MPL, DP, and KC developed the genetic and statistical strategies implemented in this work. MPL, DP, and MA performed the genetic and statistical analyses. DVJ, FS, and PWB carried out the molecular genetic studies and immunoassays. All authors participated in the design of the study and helped to draft the manuscript. All authors read and approved the final manuscript.
